# A rare case of symptomatic grossly-visible biliary intraepithelial neoplasia mimicking cholangiocarcinoma

**DOI:** 10.1186/s12957-019-1737-y

**Published:** 2019-11-11

**Authors:** Naohiro Yoshida, Takeshi Aoyagi, Yoshizo Kimura, Yoshiki Naito, Aya Izuwa, Kimihisa Mizoguchi, Kota Ishii, Yu Tanaka, Emi Ohnishi, Shun Miura, Satoshi Shimamura, Nobuhisa Shirahama, Kazuhisa Kaneshiro, Akihiro Saruwatari, Ayako Iwanaga, Yoshihiko Sadakari, Gentaro Hirokata, Toshiro Ogata, Masahiko Taniguchi

**Affiliations:** 1 Department of Surgery, St. Mary’s Hospital, Tsubukuhon-machi 422, Kurume-shi, Fukuoka 8308543 Japan; 2 Department of Pathology and Cytology, St. Mary’s Hospital, Tsubukuhon-machi 422, Kurume-shi, Fukuoka 8308543 Japan; 3 Department of Pathology, Kurume University School of Medicine, Asahi-machi 67, Kurume-shi, Fukuoka 8300011 Japan

**Keywords:** Biliary intraepithelial neoplasia (BilIN), Cholangiocarcinoma, Bile duct

## Abstract

**Background:**

Biliary intraepithelial neoplasia (BilIN) is often distinguished by what it is not: the precancerous lesions are not mass-forming, are not the cause of bile duct obstruction, and are small enough (less than 5 mm long) to evade detection by the naked eye. Here, we describe an atypical case of BilIN resembling cholangiocarcinoma (CC) that was large enough to be identified by diagnostic imaging and presented with obstructive jaundice caused by a hematoma in the common bile duct (CBD).

**Case presentation:**

A 64-year-old man presented to our hospital with upper abdominal pain and anorexia. Initial laboratory examinations revealed increased total bilirubin and a computed tomography (CT) scan revealed a dilated CBD. Gastroenterologists performed an endoscopic sphincterotomy (EST), which revealed that the cause of obstructive jaundice was a hematoma in the CBD. Enhanced CT scan and magnetic resonance cholangiopancreatography (MRCP) performed after the hematoma was drained showed improved dilation of the CBD and an enhanced wall thickness of bile duct measuring 25 × 10 mm at the union of the cystic and common hepatic ducts. A cholangioscope detected an elevated tumor covered by sludge in the CBD, and we performed an extrahepatic bile duct resection and cholecystectomy. The postoperative course was uneventful and the pathological examination of the resected tumor revealed that although the ulcerated lesion had inflammatory granulation tissue, it did not contain the components of invasive carcinoma. Many consecutive intraepithelial micropapillary lesions spread around the ulcerated lesion, and the epithelial cells showed an increased nucleus-to-cytoplasm ratio, nuclear hyperchromasia, and architectural atypia. The pathological diagnosis was BilIN-1 to -2. Immunohistochemical staining showed that S100P was slightly expressed and MUC5AC was positive, while MUC1 was negative and p53 was not overexpressed.

**Conclusion:**

We experienced an atypical case of BilIN mimicking CC that presented with obstructive jaundice caused by a hematoma in the CBD. Our case suggested that the occurrence of BilIN can be triggered by factors other than inflammation, and can grow to a size large enough to be detected by image analyses.

## Background

Cholangiocarcinoma (CC) is the second most common primary liver cancer and carries a high post-resection morbidity and mortality rate [1, 2]. Most cases of CC are detected at advanced stages as patients are usually symptom-free until the disease progresses, so the outcome of CC is generally very poor [1]. To improve this outcome, it is important to be familiar with precancerous lesions for cancer therapy. The precursor lesions of carcinoma have been advocated as adenoma in the gastrointestinal tract, intraepithelial neoplasia in uterine cervical cancer, and leukoplakia in oral cancer [3, 4]. Biliary intraepithelial neoplasia (BilIN) has been described in the World Health Organization 2010 gastrointestinal tumor classification as one of the precursor lesions of CC along with intraductal papillary neoplasm (IPNB), mucinous cystic neoplasm (MCN), and adenoma [5–7]. BilIN usually occurs in the intrahepatic bile duct and occasionally in the extrahepatic bile duct [8, 9]. Its precancerous lesions are less than 5 mm long, do not form a mass, and do not cause a bile duct obstruction [10, 11]. Because of this, detection by image analysis is usually impossible, and the diagnosis entirely depends on pathological examination [12]. Most tumors in the bile duct that are detectable by macroscopic or radiological examinations contain a malignant component, so the typical morphological characteristics, natural course, and prognosis of BilIN without CC are not well understood. Here, we describe an atypical case of BilIN resembling CC that presented with obstructive jaundice caused by a hematoma in the common bile duct (CBD).

## Case presentation

**Fig. 1:**
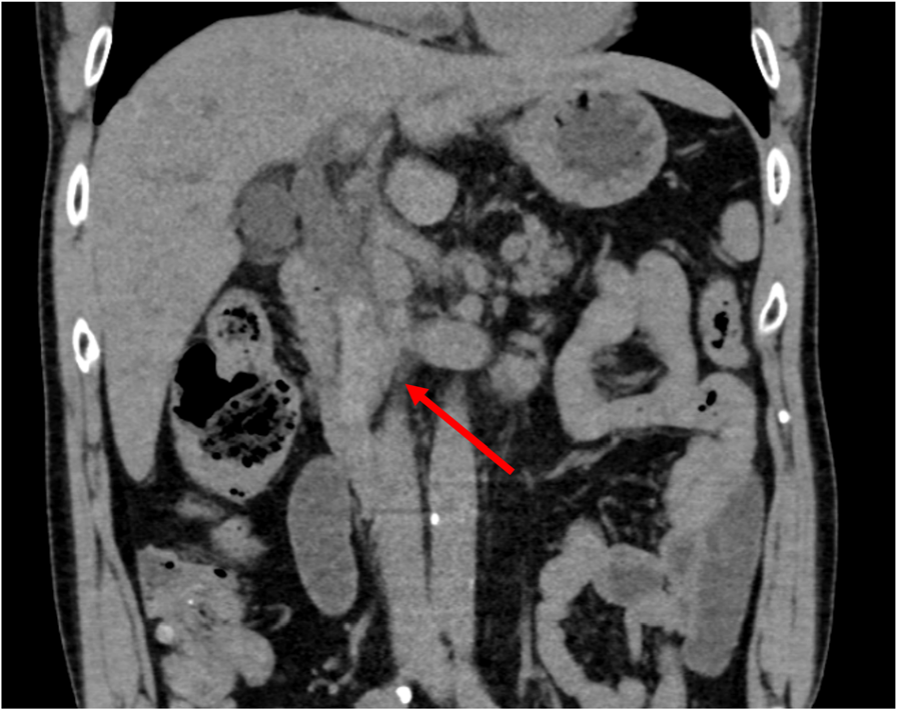
Plain CT scan image on admission. Coronal section of plain CT showing the high-density area in the CBD (arrow) and the dilation of the CBD and intrahepatic bile duct

**Fig. 2:**
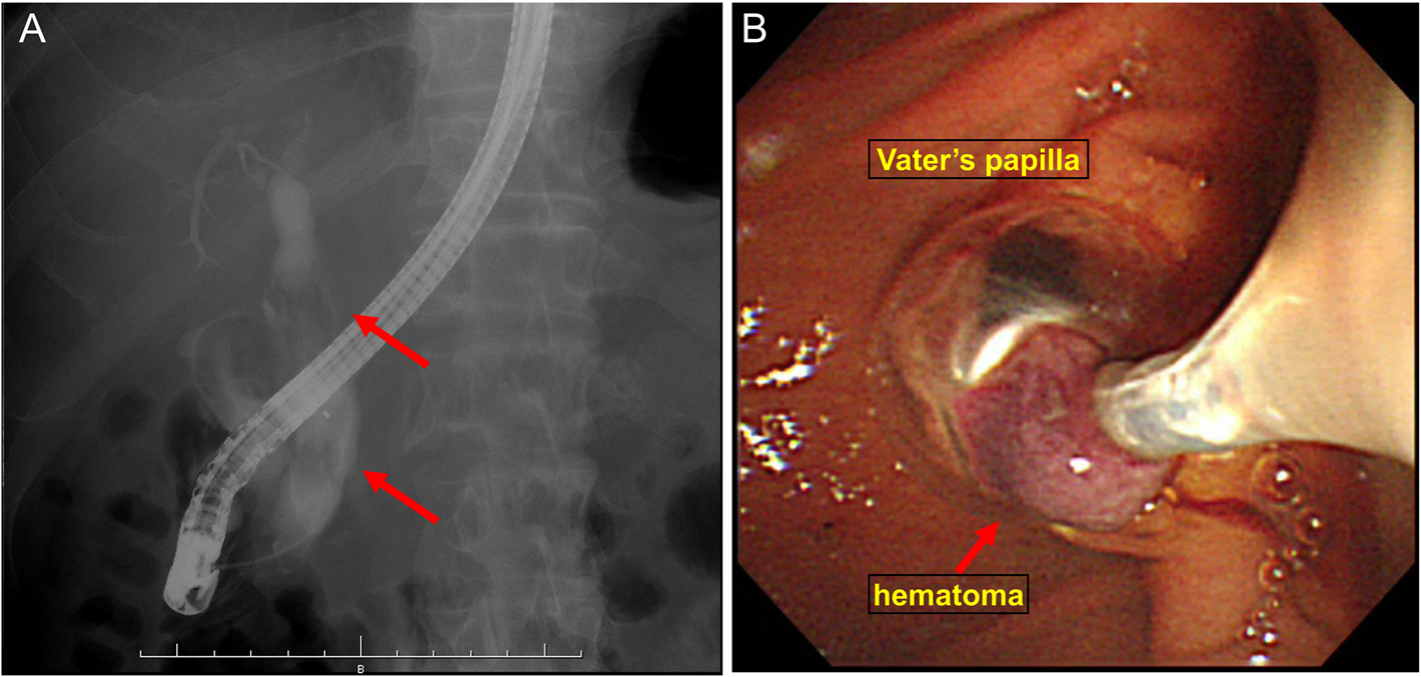
ERCP and the image of Vater’s papilla. ERCP showing a filling defect (arrows) in the CBD and a dilated CBD (**a**). EST revealed the defect was not choledocholithiasis but a hematoma in the CBD, and the hematoma was drained through the incised Vater’s papilla (**b**)

**Fig. 3:**
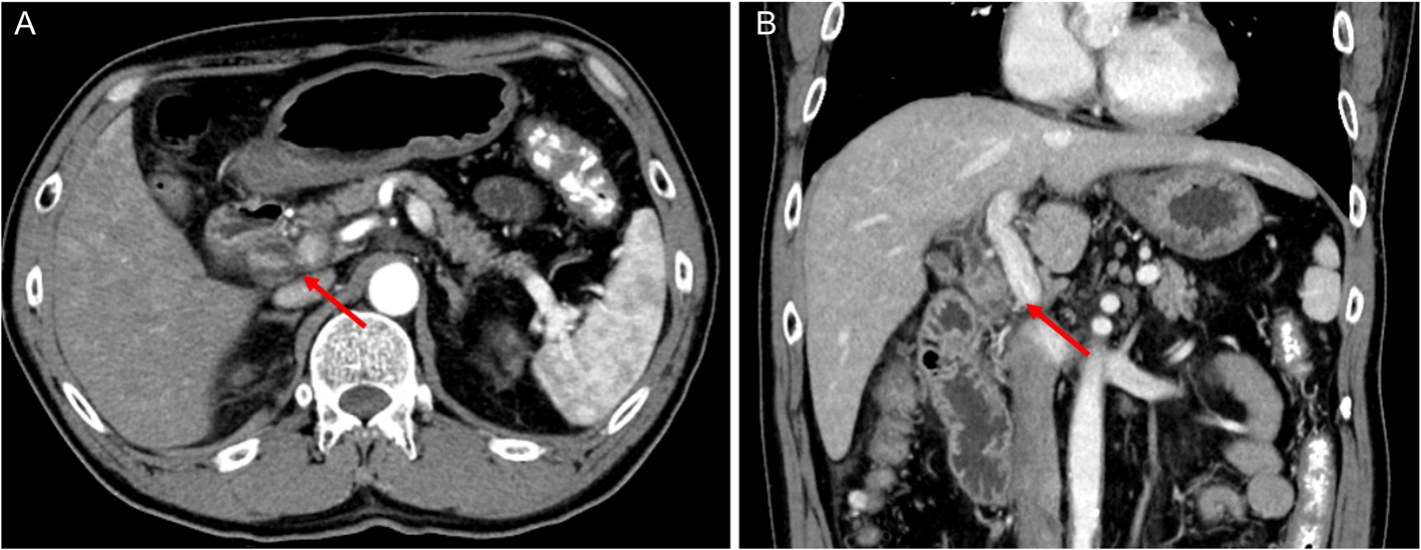
Enhanced CT scan images after EST. Enhanced CT scan showing improved dilation of the CBD and enhanced wall thickness (arrows) of the bile duct measuring 25 × 10 mm at the union of the cystic and common hepatic ducts (**a**, **b**)

**Fig. 4:**
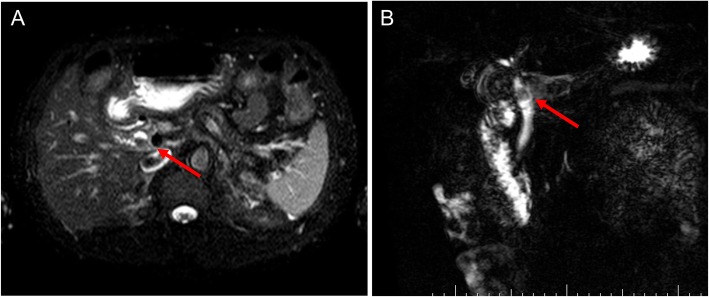
The findings of MRCP. It shows a filling defect (arrows) at the union of the cystic and common hepatic ducts (**a**, **b**)

**Fig. 5:**
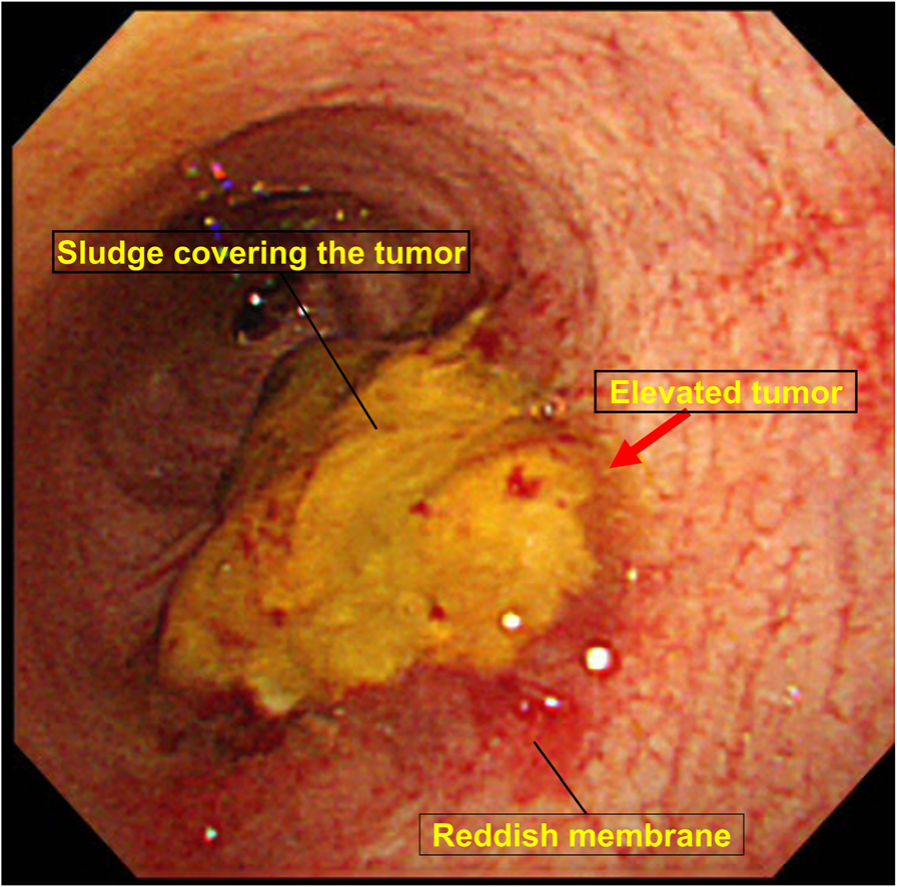
The findings of cholangioscope. It detected an elevated tumor covered by sludge in the common bile duct. The mucous membrane around the tumor showed redness and a malignant tumor was suspected

**Fig. 6:**
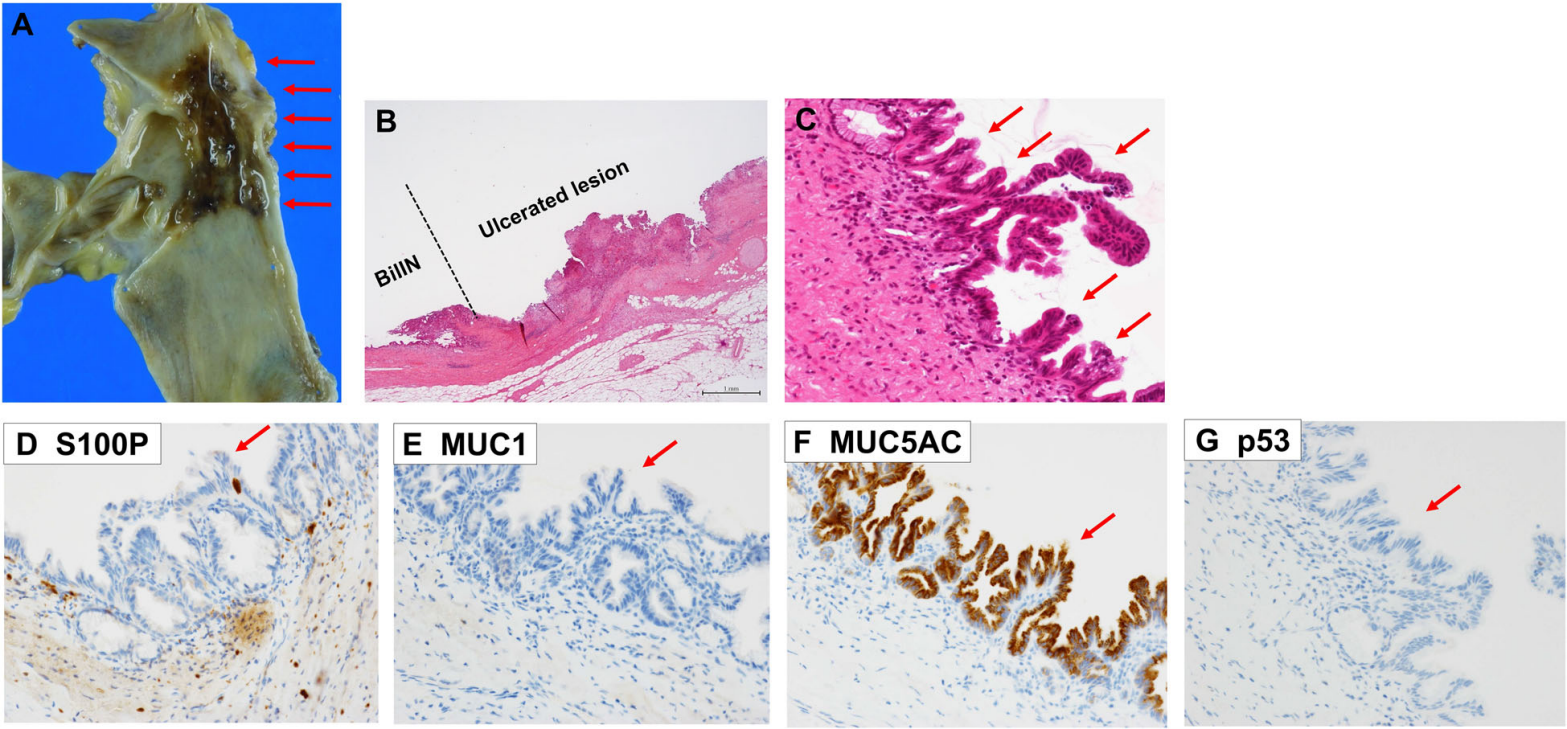
The macroscopic and pathological findings of the resected tumor. Arrows showing the irregular elevated mucosa with an ulcerated lesion of the resected tumor (**a**). The pathological examination of the resected tumor revealed that the ulcerated lesion had inflammatory granulation tissue but did not contain invasive carcinoma (**b**) (hematoxylin and eosin). Many consecutive intraepithelial micropapillary lesions spread around the ulcerated lesion (arrows), and the epithelial cells showed an increased nucleus-to-cytoplasm ratio, nuclear hyperchromasia, and architectural atypia (**c**) (hematoxylin and eosin, × 200). The pathological diagnosis was BilIN-1 to -2. Immunohistochemical staining showed that S100P was slightly expressed in the cytoplasm and MUC5AC was positive. MUC1 was negative and p53 was not overexpressed (**d** S100P, **e** MUC1, **f** MUC5AC, **g** p53) (× 200)

A 64-year-old man presented to our hospital with upper abdominal pain, jaundice, and anorexia. He had diabetes and was a social drinker but a lifetime non-smoker. Computed tomography (CT) scan revealed a dilated CBD, and acute cholangitis was suspected. The patient was referred to our hospital and admitted to the gastroenterology department for further investigation and treatment. Initial laboratory examinations revealed a white blood count (WBC) of 9770/μL, hemoglobin of 12.4 g/dl, increased C-reactive protein (CRP) of 5.47 mg/dl, total bilirubin of 7.75 mg/dl, AST/ALT of 176/281 IU/L, alkaline phosphatase of 815 IU/L, and ɤ-GTP of 132 IU/L. The serum tumor markers carcinoembryonic antigen (CEA) was within the normal range at 2.6 ng/ml and cancer antigen 19–9 (CA19–9) was elevated at 1162 U/ml. Both hepatitis B surface antigen (HBsAg) and antibodies to hepatitis C virus (anti-HCV) were negative. A plain CT scan on admission showed a high-density accumulation spreading throughout the CBD, and the entire CBD was dilated (Fig. 1). Gastroenterologists performed endoscopic retrograde cholangiopancreatography (ERCP) and endoscopic sphincterotomy (EST), during which a hematoma in the CBD was discovered. This revealed the reason for obstructive jaundice was not choledocholithiasis but the hematoma, which was subsequently drained through the incised Vater’s papilla (Fig. 2). A few days later, enhanced CT scan and magnetic resonance cholangiopancreatography (MRCP) were performed, and they showed improved dilation of the CBD and enhanced wall thickness of the bile duct measuring 25 × 10 mm at the union of the cystic and common hepatic ducts (Figs. 3 and 4). A cholangioscope detected an elevated tumor covered by sludge in the CBD (Fig. 5). The mucous membrane around the tumor showed redness and a malignant tumor was suspected. The result of the tumor biopsy revealed no malignant features in the histology, but the possibility of CC could not be denied from the macroscopic findings. We were consulted for surgical resection and performed an extrahepatic bile duct resection and cholecystectomy. Intraoperative rapid pathological diagnosis was performed, and we confirmed that the surgical margins in both the pancreatic and hepatic sides were cancer-free. The postoperative course was uneventful. The resected tumor had irregular elevated mucosa with an ulcerated lesion (Fig. 6a). The pathological examination of the resected tumor revealed that the ulcerated lesion had inflammatory granulation tissue; however, it did not contain the components of invasive carcinoma (Fig. 6b). Many consecutive intraepithelial micropapillary lesions spread around the ulcerated lesion, and the epithelial cells showed increased nucleus-to-cytoplasm ratio, nuclear hyperchromasia, and architectural atypia (Fig. 6c). The pathological diagnosis was BilIN-1 to -2. It also revealed that the BilIN-1 lesion spread through both the pancreatic and hepatic margins. Immunohistochemical staining showed that S100P was slightly expressed in the cytoplasm and MUC5AC was positive, while MUC1 was negative and p53 was not overexpressed (Fig. 6d–g).

## Discussion

BilIN has been defined by Zen et al. in 2005 as microscopic lesions of flat or low-papillary dysplastic epithelium [13, 14]. BilIN is regarded as the biliary counterpart of pancreatic intraepithelial neoplasia (PanIN) [11]. It is characterized by the pathological findings of irregular nuclei, cell dysplasia and irregular polarity in cytology [12]. The microscopic appearances of BilIN are classified as flat, pseudopapillary or micropapillary [12, 15]. BilIN is divided into three grades (BilIN-1, BilIN-2, and BilIN-3) based on the following histological features: degree of cellular atypia, nuclear pseudostratification and apical surface protrusions, and loss of cellular polarity [1]. Low-grade and high-grade dysplasia are abbreviated as BilIN-1 and -2, and in situ carcinoma as BilIN-3 [4]. BillN is considered to progress with increasing neoplastic potential, with BilIN-3 shifting to CC in the multistep carcinogenesis sequence [14]. Metaplastic changes of the epithelium of the bile duct can be induced by chronic inflammation, and patients often have a preceding chronic biliary disease like primary sclerosing cholangitis (PSC), choledocholithiasis, and pancreaticobiliary malfunction. with BilIN [12, 16].

In our case, there were two atypical findings regarding BilIN. One is that it occurred in a patient with no history of a prior biliary disease or chronic inflammation. Although the mucosa around the tumor showed redness, the range was limited to the junction of the cystic and common hepatic ducts. The other atypical finding is the large tumor size of 25 × 10 mm. Although BilIN is usually unrecognizable by macroscopy, the tumor was clearly detected in CT and could be observed as an elevated mass in the cholangioscope. To the best of our knowledge, it was larger than any other case of BilIN without CC reported in the literature so far and was therefore very difficult to differentiate from CC. These two findings in our case suggest that the occurrence of BilIN can be triggered by factors other than inflammation and that the tumor can grow to a size large enough to be detected by image analyses without shifting to CC.

The tumor was ulcerated and hemorrhaging in the CBD when the patient was referred to our hospital. In our case, obstructive jaundice caused by the hematoma in the CBD led to the detection of BilIN before progressing to CC. CC is not a major cause of hemobilia, which is present in only 3% of cases [17]. It was unclear why and when the tumor developed the ulcer and bleeding. It is also unknown that BIlIN itself causes stenosis of the bile duct and obstructive jaundice. In PanIN, the stenosis of the main pancreatic duct combined with distal ductal dilatation and obstructive pancreatitis is often observed [18]. However, PanIN itself is not considered to cause duct stenosis, because PanIN is almost flat with no intraductal proliferation, but inflammation or fibrosis [18]. The inflammation detached dysplastic epithelium and leaked pancreatic juice may cause further inflammation, fibrosis, and finally the stenosis [18]. According to this discussion, we speculate that BilIN itself also does not cause bile duct stenosis as well as PanIN, but if it combined with some inflammation, it could cause the destruction of bile duct epithelium, the stenosis of the bile duct, and obstructive jaundice.

It is important to detect precursor lesions early in cancer therapy, although the prognosis of BilIN without CC has not been clarified and a uniform treatment guideline is lacking. And unlike endoscopic resection for adenoma in the gastrointestinal tract, a minimally invasive treatment in the bile duct has yet to be developed. The only curative treatment for BilIN prior to malignant transformation is surgical resection [10]. In surgical resection for CC, an inspection of the resection margin often reveals the presence of BilIN [13]. It is well-established that resection with a cancer-free margin is crucial for curative therapy, but the clinical significance of BilIN in the surgical margin is unclear and additional resection is still controversial. Matthaei et al. reported that BilIN found in the surgical margin of biliary tract cancer resection in 53%; however, it does not require additional resection because the patients with BilIN-positive at the surgical margin and BilIN-negative did not differ significantly regarding overall survival [13]. They also reported that survival even of patients with BilIN-3 was not shorter than that of patients without any BilIN at margin [13]. Several studies also reported as well but the number of patients reported was small, so further study with a larger population and longer term is required [19, 20]..

Many molecular and genetic alternations have been found to occur and accumulate in BilIN during the multistep process of cholangiocarcinogenesis [12, 21]. The expression pattern of molecules in the tumor tissue biopsy related to cell cycle and carcinogenesis might be helpful in the preoperative diagnosis of BilIN and differentiation from CC. Representative molecules considered that can be upregulated in BilIN are p21, p53, cyclin D1, S100P, MUC1, and MUC5AC [2, 11, 12]. Some of them can be upregulated in CC too [11, 12]. On the other hand, most BilIN shows negativity for MUC2 [22]. The expression of MUC1 is increased along with the progression of histological grade in BilIN and associated with a poorer outcome in CC [2, 23]. MUC5AC expresses in the early stage of cholangiocarcinogenesis [24]. Recent studies showed the expression of S100P was increased in BilIN-2 and -3 as well as cholangiocarcinoma, and Sato et al. proposed the diagnostic algorithm of histological grading of BilIN including the expression of S100P in the cytoplasm that helps the grading of BilIN1–3 [12, 18, 25]. The expression level of p53 is very low not enough to be detected by immunohistochemistry in non-neoplastic cells because its half-life is normally very short [11]. P53 is mutated in a large number of malignant neoplasms, then mutated p53 can be detected immunohistochemically based on its overexpression [11]. Nakanishi et al. reported that p53 was significantly upregulated in BilIN-3 and CC compared with BilIN-1 and -2, and suggested that the expression of p53 might be involved in the acquisition of invasive growth in CC [11]. Such research on BilIN is crucial to elucidate the multistep carcinogenesis sequence from BilIN to CC. In our case, the tumor showed weak expression of S100P, positive MUC5AC, negative MUC1, and not overexpressed p53, which is consistent with the pathological features of BilIN-1 and BilIN-2.

## Conclusion

We experienced an atypical case of BilIN mimicking CC that presented with obstructive jaundice caused by a hematoma in the CBD. Our case suggested that the occurrence of BilIN can be triggered by factors other than an inflammatory condition and can potentially grow to a size large enough to be detected by diagnostic imaging.

## Data Availability

Data supporting the conclusions of this study are included in this published article.

## Abbreviations

Anti-HCV: Antibodies to hepatitis C virus
BilIN: Biliary intraepithelial neoplasia
CA19–9: Cancer antigen 19–9
CBD: Common bile duct
CC: Cholangiocarcinoma
CEA: Carcinoembryonic antigen
CRP: C-reaction protein
CT: Computed tomography
ERCP: Endoscopic retrograde cholangiopancreatography
EST: Endoscopic sphincterotomy
HBsAg: Hepatitis B surface antigen
IPNB: Intraductal papillary neoplasm of bile duct
MCN: Mucinous cystic neoplasm
MRCP: Magnetic resonance cholangiopancreatography
PanIN: Pancreatic intraepithelial neoplasia
PSC: Primary sclerosing cholangitis
WBC: White blood cell
